# Impact of High-Grade Patterns in Early-Stage Lung Adenocarcinoma: A Multicentric Analysis

**DOI:** 10.1007/s00408-022-00561-y

**Published:** 2022-08-21

**Authors:** Pietro Bertoglio, Vittorio Aprile, Luigi Ventura, Maria Cattoni, Dania Nachira, Filippo Lococo, Maria Rodriguez Perez, Francesco Guerrera, Fabrizio Minervini, Giulia Querzoli, Giovanni Bocchialini, Diana Bacchin, Francesca Franzi, Guido Rindi, Salvatore Bellafiore, Federico Femia, Giuseppe Salvatore Bogina, Piergiorgio Solli, Peter Kestenholz, Enrico Ruffini, Massimiliano Paci, Stefano Margaritora, Andrea Selenito Imperatori, Marco Lucchi, Letizia Gnetti, Alberto Claudio Terzi

**Affiliations:** 1 Division of Thoracic Surgery, IRCCS Azienda Ospedaliero-Universitaria di Bologna, Bologna, Italy; 2 Division of Thoracic Surgery, University Hospital of Pisa, Azienda Ospedaliero-Universitaria Pisana, Via Paradisa 1, Pisa, Italy; 3 Division of Thoracic Surgery, University Hospital of Parma, Parma, Italy; 4 St Bartholomew’s Hospital, Barts Thorax Centre, London, UK; 5 Division of Thoracic Surgery, University of Insubria, Varese, Italy; 6 Department of General Thoracic Surgery, Fondazione Policlinico “A.Gemelli” - Catholic University of Sacred Heart, Rome, Italy; 7 Division of Thoracic Surgery, Clinica Universidad de Navarra, Madrid, Spain; 8 Division of Thoracic Surgery, University of Torino, Turin, Italy; 9 Division of Thoracic Surgery, Cantonal Hospital Lucerne, Lucerne, Switzerland; 10 Division of Pathological Anatomy, IRCCS Sacro Cuore Don Calabria Hospital, Negrar di Valpolicella, Verona, Italy; 11 Division of Pathological Anatomy, University of Insubria, Varese, Italy; 12 Division of Pathological Anatomy, Fondazione Policlinico “A.Gemelli” - Catholic University of Sacred Heart, Rome, Italy; 13 Division of Pathological Anatomy, Azienda USL di Reggio Emilia-IRCCS, Reggio Emilia, Italy; 14 Division of Thoracic Surgery, Azienda USL di Reggio Emilia-IRCCS, Reggio Emilia, Italy; 15 Division of Pathological Anatomy, University Hospital of Parma, Parma, Italy; 16 Division of Thoracic Surgery, IRCCS Sacro Cuore Don Calabria Hospital, Negrar di Valpolicella, Verona, Italy

**Keywords:** Lung adenocarcinoma, TNM staging, Lung cancer, Adenocarcinoma subtypes

## Abstract

**Objective:**

The presence of micropapillary and solid adenocarcinoma patterns leads to a worse survival and a significantly higher tendency to recur. This study aims to assess the impact of pT descriptor combined with the presence of high-grade components on long-term outcomes in early-stage lung adenocarcinomas.

**Methods:**

We retrospectively collected data of consecutive resected pT1-T3N0 lung adenocarcinoma from nine European Thoracic Centers. All patients who underwent a radical resection with lymph-node dissection between 2014 and 2017 were included. Differences in Overall Survival (OS) and Disease-Free Survival (DFS) and possible prognostic factors associated with outcomes were evaluated also after performing a propensity score matching to compare tumors containing non-high-grade and high-grade patterns.

**Results:**

Among 607 patients, the majority were male and received a lobectomy. At least one high-grade histological pattern was seen in 230 cases (37.9%), of which 169 solid and 75 micropapillary. T1a-b-c without high-grade pattern had a significant better prognosis compared to T1a-b-c with high-grade pattern (*p* = 0.020), but the latter had similar OS compared to T2a (*p* = 0.277). Concurrently, T1a-b-c without micropapillary or solid patterns had a significantly better DFS compared to those with high-grade patterns (*p* = 0.034), and it was similar to T2a (*p* = 0.839). Multivariable analysis confirms the role of T descriptor according to high-grade pattern both for OS (*p* = 0.024; HR 1.285 95% CI 1.033–1.599) and DFS (*p* = 0.003; HR 1.196, 95% CI 1.054–1.344, respectively). These results were confirmed after the propensity score matching analysis.

**Conclusions:**

pT1 lung adenocarcinomas with a high-grade component have similar prognosis of pT2a tumors.

## Introduction

In 2012, the IASLC/ERS/AJCC classification of lung adenocarcinoma [1] identified different histological patterns with typical pathological features and different and well-defined prognostic behaviors, as reported in several retrospective and prospective series [2–4]. According to their prognostic impact, these patterns can be grouped in low-grade (lepidic pattern), moderate-grade (acinar and papillary pattern) and high-grade (micropapillary and solid patterns) [5]. The presence of high-grade components has a detrimental effect on prognosis both in terms of Disease-Free Survival (DFS) and Overall Survival (OS). Despite these evidences, the latest edition of TNM issued in 2018 did not take into account histological or molecular features; as a result, lung adenocarcinomas are currently considered as a single entity regardless the most represented pattern or the presence of high-grade patterns [6].

To date, the management of early-stage, lymph-node negative, NSCLC encompasses a radical surgical resection, but indication for further adjuvant therapies is still discussed given inconsistent evidence on possible benefit. Concurrently, according to the National Comprehensive Cancer Network (NCCN) guidelines [7], adjuvant therapy is usually offered in the majority of stage II patients and in some stage IB cases especially when risk factors are present. Exploring factors which might influence survival is therefore of paramount importance.

The aim of this study was to verify the influence of high-grade adenocarcinoma patterns on long-term outcomes for each pT component in a cohort of surgically resected early-stage lung adenocarcinomas.

## Methods

### Patients

All consecutive pathological stage I and II adenocarcinoma operated on between January 2014 and December 2017 in nine European Thoracic Surgery Departments were retrospectively collected. Seven Italian institutions (IRCCS Sacro Cuore don Calabria Hospital in Negrar di Valpolicella, Verona; University Hospital of Parma; University Hospital of Pisa; University Hospital of Varese; University of Sacred Heart, IRCCS Fondazione Policlinico Agostino Gemelli in Rome; IRCCS Arcispedale Santa Maria Nuova, Reggio Emilia; University Hospital of Turin), one Spanish (Clinica Universidad de Navarra) and one Swiss (Cantonal Hospital Lucerne) participated to this study.

All patients with complete information regarding pathological stage and pathological description of different patterns were included. Patients with incomplete perioperative data were excluded from the analysis.

All cases were staged according to the eighth edition International Union Against Cancer (UICC)/American Joint Committee on Cancer TNM classification.

For this study we selected all patients with pathological T1a-b-c, T2a-b, T3 N0; patients must have undergone a radical resection of the tumor with free margin with lymphadenectomy [7, 8]; radical resection was considered according to recommendations of Rami Porta et al. which included-free parenchymal margins and the absence of metastasis in the highest lymphnode retrieved [9]. Open, Video-Assisted (VATS) or Robotic-Assisted (RATS) techniques were used according to surgeons’ preferences. Patients with parietal pleura invasion (PL3) were also excluded.

Adjuvant treatments were independently discussed by the multidisciplinary team of each center.

### Histological Classification

All cases were diagnosed according to the 2012 IASLC/ATS/ERS classification [1], and all adenocarcinoma subtypes were recorded semi-quantitatively in 5% increments by pathologists in each institution. Diagnoses were reached by consensus among pathologists of the same institution that were blinded to patients’ outcomes. Patients with a component of at least 5% of solid and/or micropapillary patterns were considered as “high-grade”.

### Endpoints

The primary endpoint of this study was to evaluate the possible differences in OS and DFS of adenocarcinoma with the same pT descriptor according to the very presence of high-grade patterns, to assess any possible pattern-related influence on survival rates. Secondarily, we investigated any possible prognostic factors for OS and DFS.

### Statistical Analysis

Data were analyzed using the softwares SPSS version 26.0 for IOS (Chicago, US) and STATA 16 (Texas, US). Continuous variables were expressed in terms of mean with standard deviation (SD) or median with range, while categorical variables were expressed in terms of frequency. Two-tailed Pearson’s chi-square test was used for intergroup comparison of categorical variables while the Student’s *T*-test and ANOVA test were used for continuous variables. DFS was defined as the time from the day of surgery until the first evidence of relapse or last follow-up, and OS as the time from the day of surgery until death from any cause or the last follow-up. Recurrence was classified in local (along surgical suture line), regional (ipsilateral lung, ipsilateral chest wall or ipsilateral hilar or mediastinal lymph-node involvement) or distant. Survival and time to relapse were estimated with Kaplan–Meier, and differences in survival were determined by log-rank analysis. Pre- and post-operative prognostic factors were investigated using Cox proportional hazards regression model, using the log(−log) curves to assess the proportional hazard assumption. Multivariable analysis was performed only with variables which had at least a *p*-value ≤ 0.2 at the univariate analysis. Variables considered for univariate analysis were those clinically relevant and that better define tumor characteristics: age, sex, smoking habit, lung resection performed, surgical access used, pT descriptor, lymphovascular and pleural invasion. Adjuvant therapies were not included because, after multidisciplinary discussion, only a small proportion of patients underwent adjuvant chemotherapy. About ten events per covariate were needed to detect prognostic factors for overall and disease-free survival.

The hazard ratio (HR) and 95% confidence intervals (CI) were reported for covariates.

A propensity score matched (PSM) comparative analysis was performed to homogenize the population and to verify the results of the analysis on the entire cohort. We adjusted for potential differences between the group with high-grade patterns and the group without high-grade pattern (1:1 match). We generated a propensity score for the matched groups using logistic regression based on the patients' potential confounding baseline characteristics: age, sex, surgical approach (open vs minimally invasive), ASA score and T component. We then created a balanced cohort using an optimized performance-matching algorithm with a caliper setting of 0.02.

## Results

Six-hundred and seven patients were included in the study. Table 1 reports preoperative, perioperative, and postoperative features of patients. At final pathology report, 230 patients (37.9%) had a high-grade pattern component (either as predominant or second predominant) of which 169 solid and 75 micropapillary; among them, 14 patients had both a solid and micropapillary component. Regarding the T component was present as follow: 326 (53.7%) T1a-b-c; 233 (38.4%) T2 and 48 (7.9%) T3.

**Table 1:** Preoperative, intraoperative, and postoperative features of patients of the whole cohort and of the groups with or without high-grade component

Variable	All the cohort (607)	Non-high-grade (377)	High-grade (230)	*p*-value
Sex *n* (%)				0.075
Male	331 (54.5)	195 (51.7)	136 (59.1)	
Age at diagnosis in years (mean, range)	68.2 (41–91)	68.5 (42–91)	67.8 (41–84)	0.338
Smoking status *n* (%)				**0.005**
Never	139 (22.9)	99 (26.2)	40 (17.4)	
Former	269 (44.3)	172 (45.6)	97 (42.2)	
Active	172 (28.3)	92 (24.4)	80 (34.8)	
Respiratory comorbidities n (%)				0.712
Yes	158 (26)	96 (25.5)	62 (26.9)	
Cardiovascular comorbidities *n* (%)				0.763
Yes	365 (60.1)	228 (60.5)	137 (59.6)	
ASA score *n* (%)				0.466
1	83 (13.7)	56 (14.8)	27 (11.7)	
2	316 (52.1)	200 (53.0)	116 (50.4)	
3	162 (26.7)	95 (25.2)	67 (29.1)	
4	15 (2.5)	9 (2.4)	6 (2.6)	
FEV1% (mean, ± SD)	95.7 ± 21.6	96.7 ± 21.7	94.2 ± 21.3	0.226
DLCO % (mean, ± SD)	74.2 ± 28.7	72.2 ± 27.8	77.6 ± 29.9	0.123
Side *n* (%)				0.499
Right	359 (59.1)	219 (58.1)	140 (60.8)	
Type of resection *n* (%)				0.135
Sublobar	114 (18.7)	65 (17.2)	49 (21.3)	
Wedge resection	44 (7.2)	23 (6.1)	21 (9.1)	
Anatomic segmentectomy	79 (13.0)	49 (12.9)	30 (13.0)	
Lobectomy	469 (77.3)	301 (79.8)	168 (73.0)	
Other	15 (2.4)	15 (2.4)	11 (4.8)	
Lobectomy plus wedge resection	5 (0.8)	1 (0.3)	4 (1.8)	
Bilobectomy	5 (0.8)	0 (0.0)	5(2.2)	
Pneumonectomy	5 (0.8)	3 (0.8)	2 (0.9)	
Surgical technique *n* (%)				**< 0.001**
Open	347 (57.2)	194 (51.4)	153 (66.5)	
VATS	222(36.6)	150 (39.8)	72 (31.3)	
Robotic	38 (6.3)	33 (8.7)	5 (2.2)	
High-grade pattern *n* (%)				
Yes	230 (37.9)			
Micropapillary	75 (12.4)			
Solid	169 (27.8)			
Lymphovascular invasion *n* (%)				0.056
Present	95 (15.7)	47 (12.5)	48 (20.8)	
Visceral pleural invasion *n* (%)				**0.002**
Present	186 (30.6)	98 (26.0)	88 (38.3)	
Size of the tumor mm (mean ± SD)	23.6 ± 12.9	23.0 ± 12.7	24.7 ± 13.3	0.128
pT *n* (%)				**0.010**
pT1	326 (53.7)	224 (59.4)	102 (44.3)	
pT1a	64 (10.5)	41 (10.9)	23 (10.0)	
pT1b	163 (26.9)	116 (30.8)	47 (20.4)	
pT1c	99 (16.3)	67 (17.8)	32 (13.9)	
pT2	233 (38.4)	124 (32.9)	109 (47.4)	
pT2a	195 (32.1)	105 (27.8)	90 (39.1)	
pT2b	38 (6.3)	19 (5.0)	19 (8.3)	
pT3	48 (7.9)	29 (7.7)	19 (8.3)	

Bold values indicate statistical significance

*ASA* American Society of Anesthesiologists, *FEV1* Forced Expiratory Volume in the first second, *DLCO* Diffusion Lung Carbon Monoxide, *VATS* Video-Assisted Thoracic Surgery, *SD* Standard Deviation

We then divided the cohort according to the presence of high-grade component (Table 1).

### Overall Survival Analysis

Five-year OS of the whole cohort was 78.6% (Standard Error, SE, 0.03). Patients with a micropapillary or solid pattern component had a significantly worse overall survival compared to patients without high-grade pattern component (*p* = 0.011 and *p* = 0.023, respectively). Consequently, OS of those with a high-grade pattern component was significantly worse compared to the other patients (*p* = 0.004).

Pathological T staging significantly influenced OS (*p* = 0.050); as expected, T1a had the best survival median time (61.9 months) while T3 had the worst outcomes (50.9 months).

**Fig. 1:**
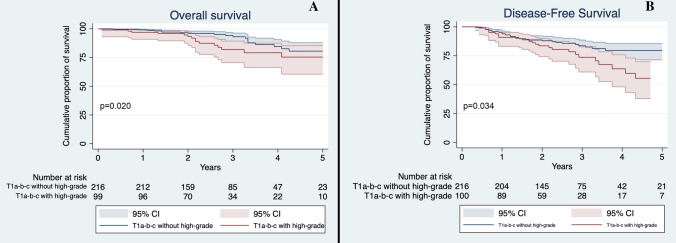
**a** Overall Survival of T1a-b-c non-high-grade and T1a-b-c high-grade in the whole cohort. Confidence interval: 95%; **b** Disease-Free Survival of T1a-b-c non-high-grade and T1a-b-c high-grade in the whole cohort. CI 95%

**Fig. 2:**
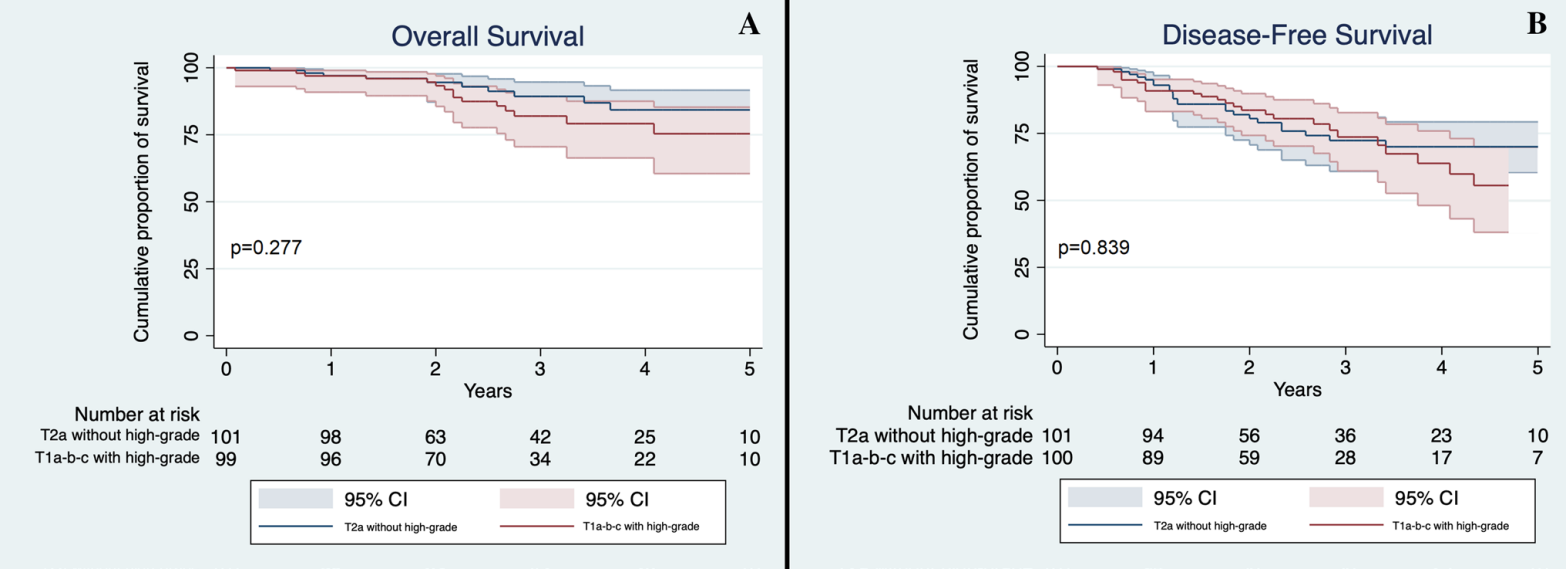
**a** Overall Survival of T2a non-high-grade and T1a-b-c high-grade in the whole cohort; **b** Disease-Free Survival of T2a non-high-grade and T1a-b-c high-grade in the whole cohort. CI 95%

**Fig. 3:**
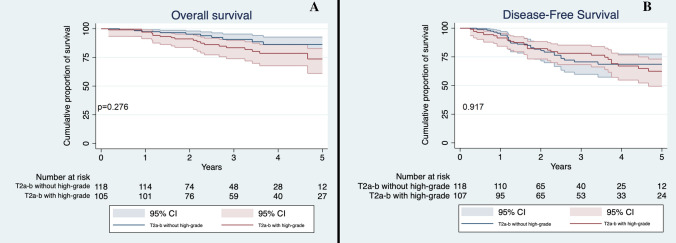
**a** Overall Survival of T2a-b non-high-grade and T2a-b high-grade in the whole cohort; **b** Disease-Free Survival of T2a-b non-high-grade and T2a-b high-grade in the whole cohort. CI 95%

**Fig. 4:**
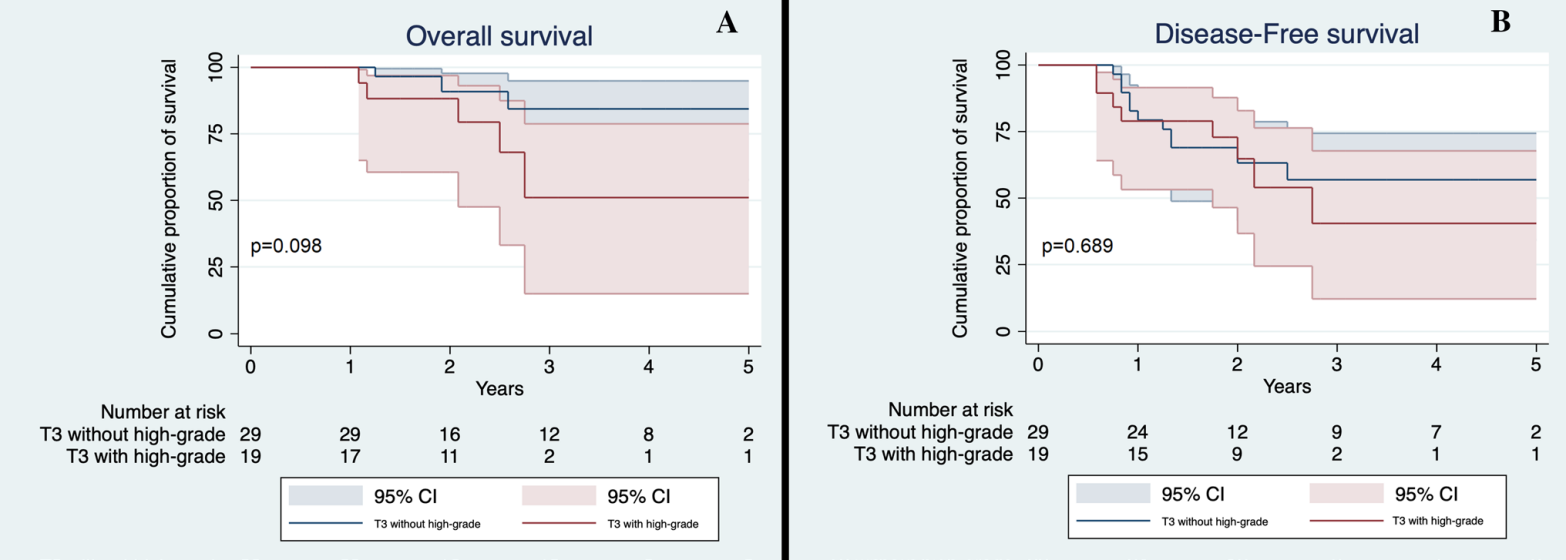
**a** Overall Survival of T3 non-high-grade and T3 high-grade in the whole cohort; **b** Disease-Free Survival of T3 non-high-grade and T3 high-grade in the whole cohort. CI 95%

In order to evaluate the possible influence of high-grade patterns on outcomes according to T descriptor, we stratified patients by their T stage and the presence of high-grade patterns. Based on this division, we appreciated a significant difference in OS (*p* = 0.011) with T1a-b-c non-high-grade accounting for the best survival median time (59.5 months, 95% CI 57.2–61.9) and T3-high-grade for the worst survival median time (43.4 months, 95% CI 31.2–54.7). We then evaluated differences between different groups: T1a-b-c non-high-grade had a significantly better survival compared to T1a-b-c high-grade (59.5 vs 56.2 months, *p* = 0.020, Fig. 1a). Conversely, T1a-b-c high-grade and T2a non-high-grade had similar OS (56.2 versus 58.7 months, *p* = 0.277, Fig. 2a). No significant differences were seen between T2 tumors with high-grade pattern and those without high-grade component (*p* = 0.276, Fig. 3a); between T2a with high-grade component and T2b without high-grade component (*p* = 0.341); between T3 with high-grade component and T3 without high-grade component (*p* = 0.098, Fig. 4a) and lastly between T2b with high-grade component and T3 without high-grade component (*p* = 0.495).

Despite a relatively small number of sublobar resections, we investigated possible differences in OS between lobectomies compared to wedge resections and lobectomies compared to all sublobar resections in the high-grade group, but the difference was not statistically significant (*p* = 0.097 and *p* = 0.232, respectively).

Univariate and multivariable analysis are reported in Table 2. At multivariable analysis age, male sex and the T stage stratified by high-grade patterns confirmed to be significant prognostic factors (*p* = 0.001; HR 1.047 95% CI 1.018–1.077, *p* = 0.002; HR 2.130 95% CI 1.314–3.451 and *p* = 0.024; HR 1.285 95% CI 1.033–1.599, respectively).

**Table 2:** Univariate and multivariable analysis of the whole cohort

	OS				DFS			
	Univariate		Multivariable		Univariate		Multivariable	
	*p*-value	HR (95% CI)	*p*-value	HR (95% CI)	*p*-value	HR (95% CI)	*p*-value	HR (95% CI)
Age (continuous variable)	***p*** = **0.001**	**1.050** **(1.021–1.080)**	***p*** = **0.001**	**1.047** **(1.018–1.077)**	***p*** = **0.005**	**1.031** **(1.009–1.053)**	***p*** = **0.018**	**1.026** **(1.004–1.048)**
Male sex (vs female)	*p* < 0.001	2.419 (1.497–3.910)	***p*** = **0.002**	**2.130** **(1.314–3.451)**	*p* = 0.062	1.388 (0.984–1.958)	*p* = 0.205	1.251 (0.885–1.769)
Smoking habit (current or former vs never)	*p* = 0.552	1.094 (0.813–1.473)			*p* = 0.821	1.027 (0.815–1.295)		
Lobectomy (vs other intervention)	*p* = 0.867	0.958 (0.579–1.584)			*p* = 0.265	0.803 (0.547–1.180)		
Minimally invasive surgery (vs open surgery)	*p* = 0.079	0.679 (0.441–1.046)	*p* = 0.103	0.650 (0.387–1.092)	*p* = 0.070	0.719 (0.503–1.028)	*p* = 0.178	0.780 (0.543–1.120)
Lymphovascular invasion (vs no)	*p* = 0.960	0.983 (0.495–1.951)			*p* = 0.937	1.019 (0.631–1.648)		
Pleural invasion (vs no)	*p* = 0.301	1.263 (0.811–1.965)			*p* = 0.396	1.167 (0.817–1.666)		
pT according to high-grade pattern	***p*** = **0.004**	**1.119** **(1.037–1.208)**	***p*** = **0.024**	**1.285** **(1.033–1.599)**	***p*** < **0.001**	**1.133** **(1.068–1.201)**	***p*** = **0.003**	**1.196** **(1.054–1.344)**

Bold values indicate statistical significance

*OS* Overall Survival, *DFS* Disease-Free Survival, *HR* Hazard Ratio, *CI* Confidence Interval

### Disease-Free Survival Analysis

Five-year DFS of the entire cohort was 67.3% (SE 0.03). According to the pattern, patients with micropapillary or solid pattern did not have a significantly worse DFS compared to patients without high-grade patterns (*p* = 0.405 and *p* = 0.172, respectively) this difference was not significant also when we compared the patterns according to their grade (*p* = 0.084).

T stage significantly influenced DFS (*p* < 0.001). When we stratified patients according to their T descriptor and the presence of high-grade patterns, we appreciated a significant difference in DFS (*p* = 0.002) with T1a-b-c non-high-grade accounting for the best mean DFS (56.2 months, 95% CI 53.5–59.0) and T3-high-grade for the worst mean DFS (36.5 months, 95% CI 25.1–48.0).

T1a-b-c without high-grade component had a significantly better DFS compared to T1a-b-c with high-grade pattern (*p* = 0.034, Fig. 1b), while the latter’s DFS was not significantly different to the T2a patients without high-grade pattern (*p* = 0.839, Fig. 2b). Finally, no significant difference was seen between T2 patients with and without high-grade component (*p* = 0.917, Fig. 3b); T3 with and without high-grade component (*p* = 0.689, Fig. 4b); T2a with high-grade component and T2b without high-grade component (*p* = 0.554); and T2b with high-grade component and T3 without high-grade pattern(*p* = 0.593).

In the high-grade group, DFS was not significantly improved in patients undergoing lobectomies compared to wedge resections (*p* = 0.513) or all sublobar resections (*p* = 0.591).

As reported in Table 2, the univariate and multivariable analysis confirmed age and pathological T stage as significant prognostic factors (*p* = 0.018, HR 1.026 95% CI 1.004–1.048 and *p* = 0.003; HR 1.196, 95% CI 1.054–1.344, respectively).

### Propensity Score Match

After performing PSM, a total of 460 patients were included in the final analysis (230 for matched pairs). The characteristics of the matched cohorts are reported and compared in Table 3.

**Table 3:** Preoperative, intraoperative, and postoperative features of patients after the propensity score matching and of the subgroups with or without high-grade component

Variable	All the cohort (460)	Non-high-grade (230)	High-grade (230)	*p*-value
Sex *n* (%)				0.924
Male	271 (59.1)	135 (58.7)	136 (59.1)	
Age at diagnosis in years (mean, range)	68.1 (41–87)	68.4 (42–87)	67.8 (41–84)	0.446
Smoking status *n* (%)				0.129
Never	97 (21.2)	57 (24.8)	40 (17.4)	
Former	193 (42.0)	97 (42.2)	97 (42.2)	
Active	148 (32.2)	67 (29.1)	80 (34.8)	
Respiratory comorbidities *n* (%)				0.635
Yes	128 (27.8)	66 (28.7)	62 (26.9)	
Cardiovascular comorbidities *n* (%)				0.909
Yes	274 (59.6)	137 (59.6)	137 (59.6)	
ASA score *n* (%)				0.551
1	59 (12.8)	32 (13.9)	27 (11.7)	
2	239 (52.0)	123 (53.5)	116 (50.4)	
3	125 (27.2)	57 (24.8)	67 (29.1)	
4	10 (2.2)	4 (1.7)	6 (2.6)	
FEV1% (mean ± SD)	95.4 ± 21.8	96.6 ± 22.2	94.2 ± 21.3	0.303
DLCO % (mean ± SD)	75.0 ± 28.7	72.7 ± 27.3	77.6 ± 29.9	0.227
Side *n* (%)				0.775
Right	278 (60.4)	137 (59.6)	140 (60.8)	
Type of resection *n* (%)				0.164
Sublobar	91 (19.8)	42 (18.3)	49 (21.3)	
Wedge resection	35 (7.6)	14 (6.1)	21 (9.1)	
Anatomic segmentectomy	62 (13.5)	32 (13.9)	30 (13.0)	
Lobectomy	349 (75.9)	181 (78.7)	168 (73.0)	
Other	14 (3.)	3 (1.3)	11 (4.8)	
Lobectomy plus wedge resection	5 (1.1)	1 (0.4)	4 (1.8)	
Bilobectomy	5 (1.1)	0 (0.0)	5 (2.2)	
Pneumonectomy	4 (0.9)	2 (0.9)	2 (0.9)	
Surgical technique *n* (%)				0.099
Open	305 (66.3)	151 (65.6)	153 (66.5)	
VATS	136 (29.6)	65 (28.3)	72 (31.3)	
Robotic	19 (4.1)	14 (6.1)	5 (2.2)	
High-grade pattern *n* (%)				n.a
Yes	230 (50.0)	0	230 (50.0)	
Micropapillary	75 (16.3)		75 (16.3)	
Solid	169 (36.7)		169 (36.7)	
Lymphovascular invasion *n* (%)				0.226
Present	77 (16.7)	31 (13.5)	48 (20.8)	
Visceral pleural invasion *n* (%)				0.071
Present	158 (34.3)	70 (30.4)	88 (38.3)	
Size of the tumor mm (mean ± SD)	24.7 ± 13.5	24.7 ± 13.8	24.7 ± 13.3	0.961
pT *n* (%)				0.426
pT1	220 (47.9)	117 (50.9)	102 (44.3)	
pT1a	45 (9.8)	21 (9.1)	23 (10.0)	
pT1b	102 (22.2)	57 (24.8)	47 (20.4)	
pT1c	72 (15.7)	39 (16.9)	32 (13.9)	
pT2	198 (43.3)	89 (38.7)	109 (47.4)	
pT2a	167 (36.3)	77 (33.5)	90 (39.1)	
pT2b	31 (6.7)	12 (5.2)	19 (8.3)	
pT3	43 (9.3)	24 (10.4)	19 (8.3)	

*ASA* American Society of Anesthesiologists, *FEV1* Forced Expiratory Volume in the first second, *DLCO* Diffusion Lung Carbon Monoxide, *VATS* Video-Assisted Thoracic Surgery, *SD* Standard Deviation, *n.a.* not applicable

**Fig. 5:**
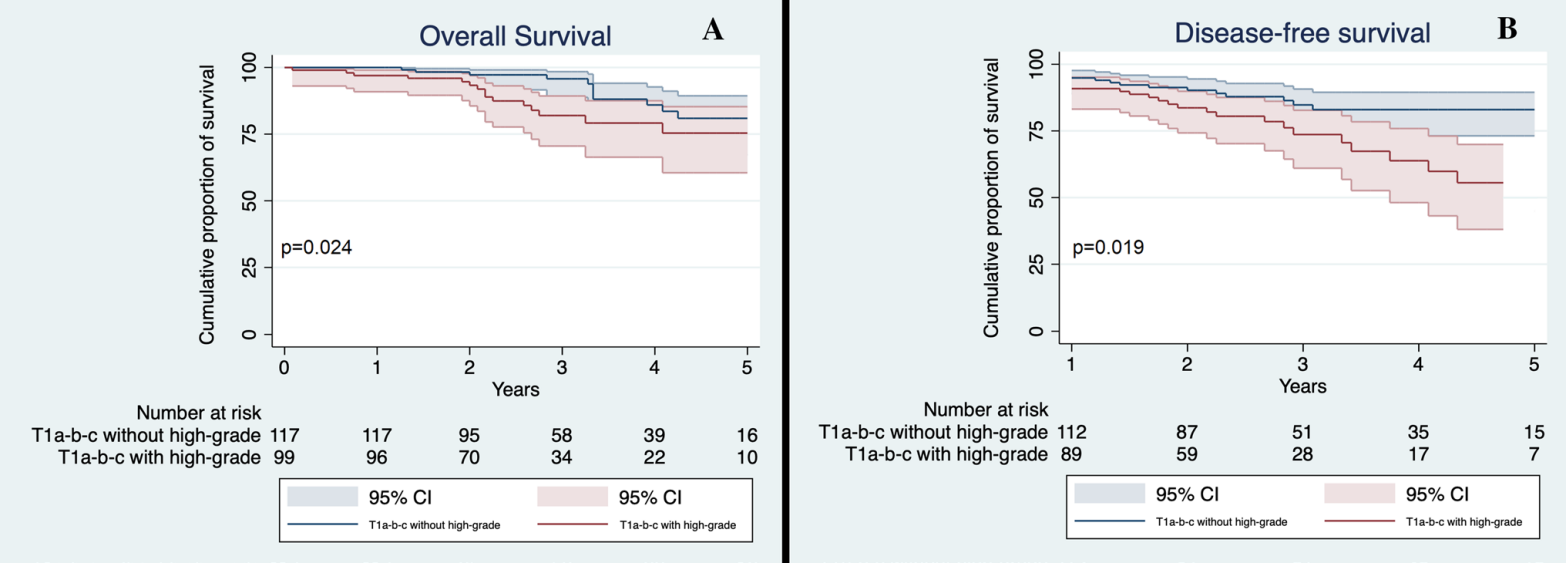
**a** Overall Survival of T1a-b-c non-high-grade and T1a-b-c high-grade in the matched cohort; **b** Disease-Free Survival of T1a-b-c non-high-grade and T1a-b-c high-grade in the matched cohort. CI 95%

The analysis of this subgroup of patients confirmed the results found in the general cohort. T1 patients without high-grade pattern had a significantly better OS and DFS compared to T1 tumors with high-grade pattern (*p* = 0.024 and *p* = 0.019, respectively, Fig. 5a and b), while no difference was seen when compared OS and DFS of T1 patients with high-grade and T2a patients without high-grade component (*p* = 0.661 and *p* = 0.890, respectively).

Univariate and multivariable analysis confirmed the prognostic value of pT according to the high-grade component (Table 4).

**Table 4:** Univariate and Multivariable analysis of the cohort obtained after Propensity Score Match

	OS				DFS			
	Univariate		Multivariable		Univariate		Multivariable	
	*p*-value	HR (95% CI)	*p*-value	HR (95% CI)	*p*-value	HR (95% CI)	*p*-value	HR (95% CI)
Age (continuous variable)	***p* < 0.001**	**1.058** **(1.027–1.090)**	*p* = **0.002**	**1.050** **(1.018–1.082)**	*p* = **0.001**	**1.042** **(1.018–1.068)**	*p* = **0.010**	**1.033** **(1.008–1.059)**
Male sex	***p* < 0.001**	**2.419** **(1.497–3.910)**	*p* = **0.025**	**1.803** **(1.078–3.015)**	*p* = **0.041**	**1.503** **(1.016–2.224)**	*p* = 0.150	1.336 (0.900–1.981)
Smoking habit (current or former vs never)	*p* = 0.903	1.019 (0.748–1.389)			*p* = 0.890	0.982 (0.765–1.262)		
Lobectomy (vs other intervention)	*p* = 0.874	0.959 (0.569–1.615)			*p* = 0.531	0.874 (0.572–1.333)		
Minimally invasive surgery (vs open surgery)	*p* = 0.103	0.622 (0.352–1.101)	*p* = 0.229	0.702 (0.394–1.249)	*p* = 0.101	0.702 (0.459–1.072)	*p* = 0.268	0.785 (0.512–1.205)
Lymphovascular invasion (vs no)	*p* = 0.784	0.904 (0.439–1.862)			*p* = 0.568	0.853 (0.494–1.473)		
Pleural invasion (vs no)	*p* = 0.253	1.310 (0.825–2.079)			*p* = 0.311	1.219 (0.831–1.790)		
pT according to high-grade pattern	*p* = **0.003**	**1.132** **(1.042–1.231)**	*p* = **0.047**	**1.091** **(1.001–1.190)**	***p* < 0.001**	**1.155** **(1.081–1.234)**	***p* < 0.001**	**1.130** **(1.056–1.210)**

Bold values indicate statistical significance

*OS* Overall Survival, *DFS* Disease-Free Survival, *HR* Hazard Ratio, *CI* Confidence Interval

## Discussion

Several non-anatomical features of NSCLC, such as EGFR/KRAS mutation [9], Spread Through Air Space (STAS) [11, 12], mitotic counts [13], genomic profile [14] have a strong influence on prognosis, but, to date, they are not considered in the TNM staging system.

In our study we analyzed the presence of high-grade patterns, namely micropapillary and solid as prognostic factor, and its impact on possible postoperative management. High-grade components are well-known and already established negative prognostic factors by several authors [2–5, 15–17]. Sica et al. [18], firstly highlighted that in metastatic lung adenocarcinoma with a non-predominant micropapillary or solid pattern, high-grade patterns were instead predominant in the metastasis tissue. Recently, a multi-institutional European group [5] explored the impact of second predominant pattern on DFS finding that the only influence was given by the presence of either micropapillary or solid pattern in the tumor. Concurrently, Yoshizawa et al. [4, 10], in two different papers, highlighted the significant prognostic impact of IASLC/ATS/ERS classification, concluding that it should have been included in the T descriptors. Similarly, Ito et al. [19] analyzed T1a and T1b lung adenocarcinoma finding that those with a smaller invasive component (namely adenocarcinoma in situ, AIS, and minimally invasive adenocarcinoma, MIA) had a significantly better DFS compared to invasive T1 adenocarcinoma. In this study we aimed to assess whether the very presence of a high-grade pattern could be considered an independent prognostic factor for OS or DFS. Our data showed that outcomes of T1a-b-cN0 lung adenocarcinomas with a high-grade component were more similar to T2a rather than T1a-b-c without high-grade components. Conversely, no further differences were seen comparing T component according to the presence of a high-grade subtype, suggesting that the tumor dimensions define its aggressivity. We hypothesize that in T1 tumors the presence of a high-grade pattern could cause a difference in survival, like visceral pleura invasion (PL1 or PL2). To the best of our knowledge, this is the first study reporting significant differences in survival rates between tumors with the same T component but different grade histological subtypes.

These results might have important clinical implications: a risk stratification based on the presence of a high-grade pattern, might allow a more accurate perioperative management. As a matter of fact, to date, NCCN guidelines [7] recommend adjuvant therapy in case of stage IB NSCLC (T2aN0) with particular risk factors, such as poorly differentiated tumors, vascular invasion, wedge resection, visceral pleural involvement, and incomplete lymph-node sampling. Consistently, Yoshiya [20] suggested a possible benefit of adjuvant therapy in case of micropapillary or solid patterns of small-sized (< 2 cm) lung adenocarcinoma considering the presence of these high-grade patterns as a risk factor for a worse OS and DFS; the same conclusions were shared by Zhang [21]. On the other hand, high-grade patterns generally showed a good response to chemotherapy, even though results on OS and DFI were inconsistent. In a large series of patients taken from previous clinical trials, Tsao [22] reported a significant impact of adjuvant treatments on DFS, but not on OS; similar conclusions were drawn by Luo and coworkers [23] in a subset of high-grade predominant pattern stage IB adenocarcinomas. Lastly, investigating the prognostic role of adenocarcinoma subtypes in stage IB patients, Ma [24] reported a significantly better DFS of adjuvant chemotherapy only in patients with high-grade predominant pattern. Conversely, Whang et al. [25] found a significant impact both on OS and DFS in a group of stage IA micropapillary adenocarcinomas. In our study, since only 20 patients (3.3% of our cohort) underwent adjuvant chemotherapy, we did not perform any analysis on its impact on OS or DFS as no significant conclusions would have been robust enough. Nevertheless, we might speculate that a preoperative diagnosis of high-grade pattern could at least influence the surgeons’ choice preferring a larger and more radical resection, such as a lobectomy, rather than sublobar resections.

Recently, sublobar resections were proposed as standard of care in tumors smaller than 2 cm, while for bigger tumors lobectomy or multi-segmental resections are still the standard of care [26, 27]. Similarly, the presence of Spread Through Air Space (STAS), which is more frequent in high-grade adenocarcinomas, has been verified to be a risk factor for early recurrence and worse survival in case of limited resections compared to lobectomy [11]. Although our series was not intended to verify differences according to the extent of the resection, we investigated possible differences in outcomes. No differences in OS and DFS were seen neither in the whole cohort nor in the subgroup of high-grade component according to a lobar or sublobar resection; moreover, lobectomy compared to other resections was not a significant prognostic factors in univariable and multivariable analysis.

Although the present study was based on a large multi-institutional database, it presents some limitations that might have influenced the quality of data and eventually the results. The major limitations are the retrospective character of the study; the missing data (e.g.: in 29% of cases data on lymphovascular invasion were missing; data on mutational status and targeted therapies were not available for most patients) and the absence of an external review or concordance analysis regarding the analysis of pathological specimens at each independent institution.

In conclusion, micropapillary and solid patterns confirm their detrimental effect on OS and DFS. The results of our study suggest that patients affected by a T1a-b-c adenocarcinoma with a high-grade pattern have similar survival outcomes of pT2a tumors. On the other hand, the effect of high-grade pattern on larger tumors seems to be marginal. According to these data, we believe that patients affected by T1a-b-c lung adenocarcinoma with a high-grade histological component should be considered for a more careful perioperative management encompassing anatomical resections and possible adjuvant therapy and/or closer surveillance.

Prospective larger studies are needed to validate these findings and properly evaluate benefit of postoperative treatment or different surveillance management in these patients.
